# Lateral sclerosis in amyotrophic lateral sclerosis is an upper motor neuron distal axonopathy (“dying back”): a clinical-neuropathological study

**DOI:** 10.1007/s00401-026-03072-9

**Published:** 2026-10-09

**Authors:** Jianguo Liu, Kailee Ong, Alex Meng, Gorazd B. Stokin, Jeffrey A. Loeb, John Ravits, Fei Song

**Affiliations:** 1 Department of Neurology and Rehabilitation, The University of Illinois at Chicago, NPI North Bldg., Room 174N, M/C 796, 912 S. Wood Street, Chicago, IL 60612 USA; 2 ALS and Neuromuscular Translational Research University of California, San Diego, CA 92093 USA; 3 Institute for Molecular and Translational Medicine, Faculty of Medicine and Dentistry, Palacký University, Olomouc, Czech Republic; 4 Department of Neurology, Gloucestershire Royal Hospital, Gloucestershire Hospitals NHS Foundation Trust, Gloucester, UK

**Keywords:** Amyotrophic lateral sclerosis, Corticospinal tracts, Axon neurodegeneration, Dying back, Myelin loss, Neuroinflammation

## Abstract

The hallmark neuropathological feature of upper motor neuron (UMN) degeneration in amyotrophic lateral sclerosis (ALS) is the “sclerosis” of the lateral corticospinal tract (CST)—the major axonal pathway connecting UMNs to lower motor neurons (LMNs). However, few studies since Charcot’s original descriptions in the nineteenth century have directly correlated clinical UMN phenotypes with corresponding neuropathological findings. We examined the pathology of the lateral CST at the cervical, thoracic, and lumbar levels of the spinal cord from ALS patients with predominantly UMN (UMN-p) phenotypes and compared it with that in patients with predominantly LMN (LMN-p) phenotypes. We assessed myelin loss, axonal degeneration, neuroinflammation, and TDP-43 pathology. Spinal cords from UMN-p ALS cases exhibited pronounced myelin loss in the lateral CST compared to LMN-p cases and controls, consistent with clinical phenotyping. Quantitative analysis revealed significant axonal loss across all axon diameter ranges in UMN-p ALS, which was more severe than in LMN-p ALS. Importantly, axonal loss was more pronounced in the lumbar than in the cervical spinal cord. We identified a distinct population of foamy, inflammatory microglia localized to regions of severe myelin and axonal loss in UMN-p ALS. TDP-43 was measurably increased in the UMN axons of the lateral and ventral CSTs. In summary, “lateral sclerosis” of ALS correlates to clinically imputed UMN degeneration. It consists of a combination of myelin loss, distal greater than proximal axonal degeneration, inflammatory microglial activation within the CSTs, and abnormally increased axonal TDP-43. The length-dependent gradient of motor axonal loss supports a "dying-back" process. A detailed comparison of “dying-forward” and “dying-forward” is provided in the Discussion. The association of loss of axons and myelin with abnormal microglial activity shows glio-axonal interactions in UMN axon degeneration, the latter CST (lateral sclerosis) across all three spinal cord levels in the ALS UMN-p samples but does not establish a causal relationship.

## Introduction

The original descriptions of “lateral sclerosis” in amyotrophic lateral sclerosis were by Charcot in the 1860s, who noted sclerosis as a pathological change in the lateral columns of the spinal cords of patients dying from progressive motor deterioration in addition to loss of neurons in the anterior horn [17, 18]. The lateral columns were subsequently recognized to be the lateral corticospinal tracts (CST), which carry axons from upper motor neuron (UMNs) cell bodies in the motor cortex to the lower motor neurons (LMNs) in the anterior horns of the spinal cord [24, 31, 69]. UMN axons descend from the cortex to the cervical-medullary pyramids, where about 75–90% decussate to form the lateral CST, and descend in the lateral funiculus. The remaining 10–25% of axons remain uncrossed and descend ipsilaterally in the ventral CST [23, 44, 45, 56, 59]. Charcot originally postulated that the hallmark lateral sclerosis was the primary driver of ventral horn cell degeneration—hence, the sclerosis was considered amyotroph*ic*. The descriptions were done at a time when the histological techniques were crude compared to modern techniques. Much of the research attention today in the ALS field is neuron-centric, focusing on motor neuron degeneration and TDP-43 abnormalities, the hallmarks of ALS neuropathologically being TDP-43 translocation from the nucleus and cytoplasmic accumulation and phosphorylation. Only relatively recently has research increased in axon functions [19, 54, 60] and to axonal transport and accumulation of TDP-43 [3, 10, 11, 43, 49, 57]. The most direct way to study UMN axons in ALS is to study the lateral sclerosis neuropathologically—the relationship between ALS biology and neuropathology is not yet understood, and it may be different for UMNs than LMNs. In this study, we characterize lateral sclerosis in the lateral CST. As the point of departure, we compared the tracts between two ends of the phenotypic clinical spectrum—spinal cords from patients who displayed predominantly UMN clinical deficits (UMN-p ALS)—and spinal cords from patients who displayed predominantly LMN clinical deficits (LMN-p ALS). We assessed myelin loss, axonal degeneration, neuroinflammation, and TDP-43 pathology.

## Materials and methods

### ALS patient population and tissues

All nervous tissues were acquired by way of an Institutional Review Board (IRB) and Health Insurance Portability and Accountability Act (HIPAA) compliant process. The acquisition and de-identification of postmortem tissues were approved by either the Benaroya Research Institute (2003–2011) or the University of California, San Diego (after 2011) IRBs, by relevant guidelines and regulations according to the Declaration of Helsinki. Subsequent use of de-identified postmortem tissues was by standard federal policies and regulations and is not considered Human Subjects research. Formalin-fixed and paraffin-embedded (FFPE) cervical, thoracic and lumbar spinal cords from sporadic ALS (sALS) patients. All patients had been classified during life by one clinical neurologist (JR) based on clinical examination and clinical signs. Signs imputing UMN degeneration were loss of fine skilled movements, spasticity, hyperreflexia, and pathological reflexes, and signs imputing LMN degeneration were weakness, atrophy, and fasciculation at disease onset. The contributions of UMN and LMN degenerations to the overall clinical deficits were recorded in a five-point scale: UMN ≫ LMN, UMN > LMN, UMN = LMN, UMN < LMN, and UMN ≪ LMN [2, 31]. The extremes (UMN ≫ LMN, *n* = 6; UMN ≪ LMN, *n* = 7) were chosen for most pathological studies and are hereafter referred to as UMN-p and LMN-p, respectively. “Typical” ALS were the three intermediate grades (UMN > LMN, UMN = LMN, or UMN < LMN, *n* = 6), and control patients (*n* = 13) with no pathological evidence for neurological disease (Table 1). The UMN-p group could arguably be classified as primary lateral sclerosis (PLS) were it not for the rapid clinical course and sometimes the co-existence of some LMN signs. The LMN-p would be called progressive muscular atrophy (PMA) by some clinical neurologists. Patient demographics are stated for convenience (Table 1).

**Table 1:** Information for sporadic ALS patients and control patients

No.	Primary diagnosis	Patient autopsy #	Motor phenotype: UMN-p or LMN-p clinical signs	Sites of onset	Disease course (years)	Age	Sex	Laboratories where studied
1	sALS	119	UMN ≫ LMN	Arm	2.5	46	M	UIC and UCSD
2	sALS	25	UMN ≫ LMN	Arm	0.9	53	M	UIC and UCSD
3	sALS	46	UMN ≫ LMN	Arm	5	51	F	UIC
4	sALS/C9orf72	82	UMN ≫ LMN	Bulbar	2	56	F	UIC and UCSD
5	sALS/C9orf72	117	UMN ≫ LMN	Bulbar	1	66	F	UIC
6	sALS	12	UMN ≫ LMN	Diffuse	1.25	60	M	UIC
7	sALS	108	LMN ≫ UMN	Leg	9	69	M	UIC and UCSD
8	sALS	109	LMN ≫ UMN	Leg	NA	49	M	UIC
9	sALS	112	LMN ≫ UMN	Leg	4	54	F	UIC
10	sALS	29	LMN ≫ UMN	Leg and foot	3.0	77	F	UIC
11	sALS	107	LMN ≫ UMN	Trunk	0.75	74	M	UIC and UCSD
12	sALS	22	LMN ≫ UMN	Respiratory and hand	3.25	72	M	UIC
13	sALS	127	LMN ≫ UMN	Respiratory and right arm	1.5	67	M	UCSD
14	sALS	32	LMN > UMN (typical)	Respiratory and trunk	1.5	71	M	UCSD
15	sALS	33	LMN = UMN (typical)	Arm	6.5	54	M	UCSD
16	sALS/FTD	93	UMN > LMN (typical)	FTD/bulbar	1.75	71	M	UCSD
17	sALS	128	LMN > UMN (typical)	Right foot	2	69	F	UCSD
18	sALS	132	LMN = UNM (typical)	Right arm	12	64	F	UCSD
19	sALS/FTD	134	UMN > LMN (typical)	Leg and bulbar	1.9	78	F	UCSD
20	Ctrl	20	NA	NA	NA	84	M	UIC
21	Ctrl	40	NA	NA	NA	67	M	UIC and UCSD
22	Ctrl	77	NA	NA	NA	68	M	UIC and UCSD
23	Ctrl	4	NA	NA	NA	75	M	UIC
24	Ctrl	37	NA	NA	NA	57	M	UIC and UCSD
25	Ctrl	65	NA	NA	NA	82	M	UIC
26	Ctrl	115	NA	NA	NA	94	M	UIC
27	Ctrl	76	NA	NA	NA	76	F	UIC and UCSD
28	Ctrl	78	NA	NA	NA	58	F	UIC
29	Ctrl	19	NA	NA	NA	80	F	UIC
30	Ctrl	44	NA	NA	NA	80	F	UCSD
31	Ctrl	83	NA	NA	NA	63	F	UCSD
32	Ctrl	103	NA	NA	NA	92	F	UCSD

### Histopathology

Sections of FFPE human spinal cord blocks were cut transversely at 5 µm thickness, dewaxed with xylene, and hydrated through a graded series of alcohol solutions. The sections were then stained with Luxol Fast Blue-Periodic Acid Schiff (LFB-PAS) (Poly Scientific, Bay Shore, NY) for the presence of myelin according to manufacturer’s instruction [62, 63]. 20 × digital images of intact spinal cords were obtained with a Leica DM5500B microscope (Leica Microsystems Inc., Wetzlar, Germany)*.*

### Immunofluorescence

To examine axonal degeneration and myelination in ALS and control patients, the following primary antibodies were used to examine the expression of neurofilament heavy chain and myelin basic protein in the white matter of spinal cords; anti-Neurofilament heavy chains (1:50, mouse monoclonal; clone RT97, DSHB, Iowa City, Iowa) and anti-myelin basic protein (MBP) (1:200, Cat. No. A0623, Rabbit polyclonal, Dako). The sections were then incubated with goat anti-rabbit antibody tagged with Alexa-488 (1:250, Code: 111-545-144, Jackson Immuno Inc.) and biotinylated-goat anti-mouse antibody (1:250, Code: 115-065-062, Jackson Immuno Inc.) for 2 h followed by streptavidin conjugated with Alexa-594 for 1 h (1:500, Code: 016-580-084, Jackson Immuno Inc.). The slides were mounted with antifade mounting medium (Cat. No. H-1200, Vector Laboratories, Burlingame, CA).

Tissue staining for microglia and motor neurons was performed using Iba-1 (rabbit polyclonal 1:500, Cat. No. 019-19741 Wako Inc. Richmond, VA), and Neurotracer™ 530/560 red, fluorescent Nissl stain (1:500, Cat. No. N21482, Fisher Scientific, USA), respectively. After antigen retrieval (10 mM EDTA, pH6.0), nonspecific binding sites were blocked for one hour at room temperature with blocking solution (5% normal goat serum, 5% BSA and 0.05% Triton X-100 in TBS, pH7.4); the primary antibody was diluted in the blocking solution and added onto the sections overnight at 4 °C, and the sections were incubated with goat anti-rabbit Alexa Fluor 488 (1:500, Invitrogen, Carlsbad, CA). The sections were then counterstained with Neurotracer 530/615 (1:500, Cat. No. N21482, Fisher Scientific, USA), treated with 0.3% Sudan Black for minimizing autofluorescence, and mounted with antifade mounting medium (Cat. No. H-1000, Vector laboratories, Burlingame, CA) [22].

To examine axonal TDP-43, 6 µm-thick axial cross-sections of FFPE thoracic spinal cords from sALS and control (Table 1, UCSD site) patients were dual-immunolabeled with anti-phosphorylated Neurofilament Heavy (pNF-H) (Chemicon #AB5539, 1:1000) and anti-TDP-43 (ProteinTech #10782-2-AP, 1:3000). The pNF-H antibody has previously been validated to target myelinated and unmyelinated axons in human tissue [7, 30]. The primary antibodies were visualized with AlexaFluor 568 anti-rabbit IgG (Invitrogen #A10042, 1:250) and 488 anti-chicken IgY (Invitrogen #A78948, 1:500). Coverslips were mounted on the slides using ProLong Gold Antifade Media with DAPI (Invitrogen #P36935) and imaged using identical exposure settings (DAPI: 21.067 ms; FITC: 355.26 ms, TRITC: 227.752 ms) on the Olympus VS200 Slide Scanner at the UCSD Microscopy Core.

### Immunohistochemistry

Identification of microglia was performed using antibodies specific to human CD68 (mouse IgG1, 1:20, catalog no. N1577; DAKO, Carpinteria, CA). Astrocytes were labeled with anti-glial fibrillary acidic protein (GFAP) antibodies against human (rabbit polyclonal, 1:100; Cat. No. Z0334, lot 00076541, DAKO). The primary antibodies were diluted in blocking solution (10% normal goat serum, 0.05% Triton X-100 in phosphate-buffered saline) overnight at 4 °C followed by incubation with goat anti-rabbit Alexa Fluor 488 (1:100; Invitrogen, Carlsbad, CA). For CD68 immunostaining, biotin-conjugated goat anti-mouse was used as a secondary antibody; the signal was amplified using a tyramide signal amplification kit (1:250; Invitrogen) following the manufacturer’s instructions [62].

### Quantitative image analysis

The digital intact spinal cord images were obtained with a Leica DM5500B fluorescence microscope (Leica Microsystems Inc., Wetzlar, Germany) and scanned automatically with the multiple channel option in a Q (Imaging) cooled CCD camera (SN: Q36526, Canada). In the only step involving operator discretion, all image parameters including auto-expose setting, target intensity, binning, camera area, background subtraction, shading correction, color tab, special tab and temperature were first set using control patient tissue, and the same setting used for all samples imaged. The image size was the same for all images. The specific fluorescent labeled molecules in the lateral CST, dorsal column (DC) and laminae zone VIII&IX of the ventral horns were analyzed with Metamorph Software 7.8 with color channel separation.

### Axon quantifications

Four regions of interest (ROIs) were randomly selected from the neurofilament (NF, RT97) channel on both the right and left sides of the lateral CST, or three ROIs from bilateral DC, at each spinal cord level per patient. Axon size thresholds were determined for each blinded ROI using Metamorph image analysis software, and axon sizes were quantified as pixel area. Raw data were exported to Microsoft Excel and analyzed using the Excel Data Analysis ToolPak. Axon frequency histograms were generated from raw axon pixel area data using bin ranges of 20–500 pixels. Repeatability was assessed within each patient group (*n* = 6–10).

In detail, quantification of axons (NF RT97) was performed in the Metamorph (Molecular Devices Inc. Downington, Pennsylvania, USA) software using a custom script. The original mono red color (RT97) images were used and the background fluorescence eliminated and then exported data automatically to Excel for generating the given data on density of positive axon for each image. Briefly, two thresholds for bright and dark of positive RT97 staining were determined by an average gray through 20 bright and dark objects and recorded in a notebook for each image. Pixels with signals above thresholds constitute axons of interest. To eliminate background noise, dark objects were defined as total area between 20 and 1E+00.8 pixels, shape factor between 0.7 and 1, and average gray between 0 and 1E+00.7. Dark objects are those with total area between 10 and 1E +00.8, shape factors between 0.7 and 1, and average gray between 0 and 1500 pixels. Threshold of the images for dark objects, integration morphometry analysis and log data were imported into Excel. All selected regions around dark objects were created and painted using display-graphic, paint regions, and choice insider regions. To isolate dark objects, the image threshold was adjusted to exclude bright objects, leaving behind halo-like structures. An average gray value threshold was then applied to remove these halo-objects. Then, integrated morphometry analysis for dark objects and log data were imported into Excel. Finally, the lists of bright and dark objects were combined.

Raw axon size data were exported from Metamorph software and collected in Excel format for analysis. The data were analyzed using the “Data Analysis” tool integrated into Excel, selecting the Histogram function from the list of available options. In the histogram dialog box, raw axon size values (pixel area) were entered along with predefined bin ranges. The Excel file above contains values for axon number, total area of axon, average gray of axon, total gray of axon, and shape factor of axon. The histograms were generated using the total area of axon to indicate the frequency of axons within certain diameter ranges for each image. For the average axon frequency in each axon diameter range, the mean of axon frequency from four randomly selected regions (within the lateral CST) or three regions (within the DC) in the spinal cord from each patient was calculated.

Axon size distributions were plotted as histograms using pixel area bins of 20, 40, 60, 80, 100, 120, 140, 160, 180, 200, and 500 pixels (Fig. 2A), corresponding to axon diameters of approximately 3.2, 5.6, 6.5, 7.2, 7.9, 8.5, 9.1, 9.7, 10.2, and16.4 µm, calculated using the conversion formula: 2 × √((0.642 × pixel area) / 3.14) (Fig. 2B–D). Mean axon pixel area values were calculated for each bin range for UMN-p ALS (*n* = 6), LMN-p ALS (*n* = 6), and controls (*n* = 10). Axon frequency histograms were generated separately for each spinal cord level (cervical, thoracic, and lumbar) across control, UMN-p ALS, and LMN-p ALS groups.

ALS group values at each spinal level were normalized to the corresponding control values and expressed as percentages (control = 100%). In the resulting Excel histograms, mean normalized values for each axon diameter were analyzed. Normalized UMN-p and LMN-p ALS values were compared between axon diameter range of 3.2–16.4 µm. Logarithmic (log_2_) scaling was applied to facilitate comparisons across wide value ranges. These values were calculated for motor axons (lateral CST) and sensory axons (DCs) in the UMN-p ALS spinal cords and compared with corresponding axon diameter in LMN-p ALS spinal cords after normalizing to the respective axon size in control patients.

A total of 48 (12 ALS patients) and 40 (10 control patients) randomly selected regions within the lateral CSTs from each spinal cord were analyzed at each spinal cord level (cervical, thoracic, lumbar). A total of 36 (from 12 ALS patients) and 30 (from 10 control patients) randomly selected regions within the DC at each level of spinal cord were analyzed.

### Microglial quantifications

To quantify microglia in the lateral CST, a manual count was completed blindly. A randomly selected region within the left and right lateral CSTs at each level section of the spinal cord was sampled. The size of the randomly selected region was a circle of 96 pixels in diameter. Using the Metamorph ‘manual count object’ function, the microglia were quantified in each sampled lateral CST. Criteria for microglia included being circular and either a ring or filled in shape with the size of microglia not having an impact on the decision. The average number of microglia per lateral CST (left and right sides) was calculated at each level of the spinal cord for each patient.

### Axonal TDP-43 quantifications

Image analysis was performed on QuPath-0.6.0-rc4 [5] and Fiji 1.54p. The boundaries of the regions of interest (ROI)—lateral CST, ventral CST, and DC—were approximated and hand-annotated on each image. To calculate the frequency of axonal TDP-43 in these white matter tracts, the number of TDP-43 signals that occurred in the axoplasm (i.e., co-localized with pNF-H) was then manually counted and normalized to the area of each ROI. For the gray matter, the ventral to the posterior edge of the central canal was manually segmented and extrasomatic TDP-43 signals were quantified. The putative signals were then filtered based on their pNF-H intensity and morphology to exclude signals unassociated with axons.

### Statistical analysis

For axon quantifications, statistical differences among the three groups were assessed using two-way ANOVA, while comparisons between the two ALS groups were performed using Student’s *t* test. For axonal TDP-43, statistical significance was assessed using the Kruskal–Wallis test or the Mann–Whitney test. Significance thresholds were set at *p* < 0.05 and *p* < 0.001.

## Results

### Myelin loss is severe in the lateral CST in spinal cords from ALS patients with UMN-p symptoms but not LMN-p symptoms

**Fig. 1:**
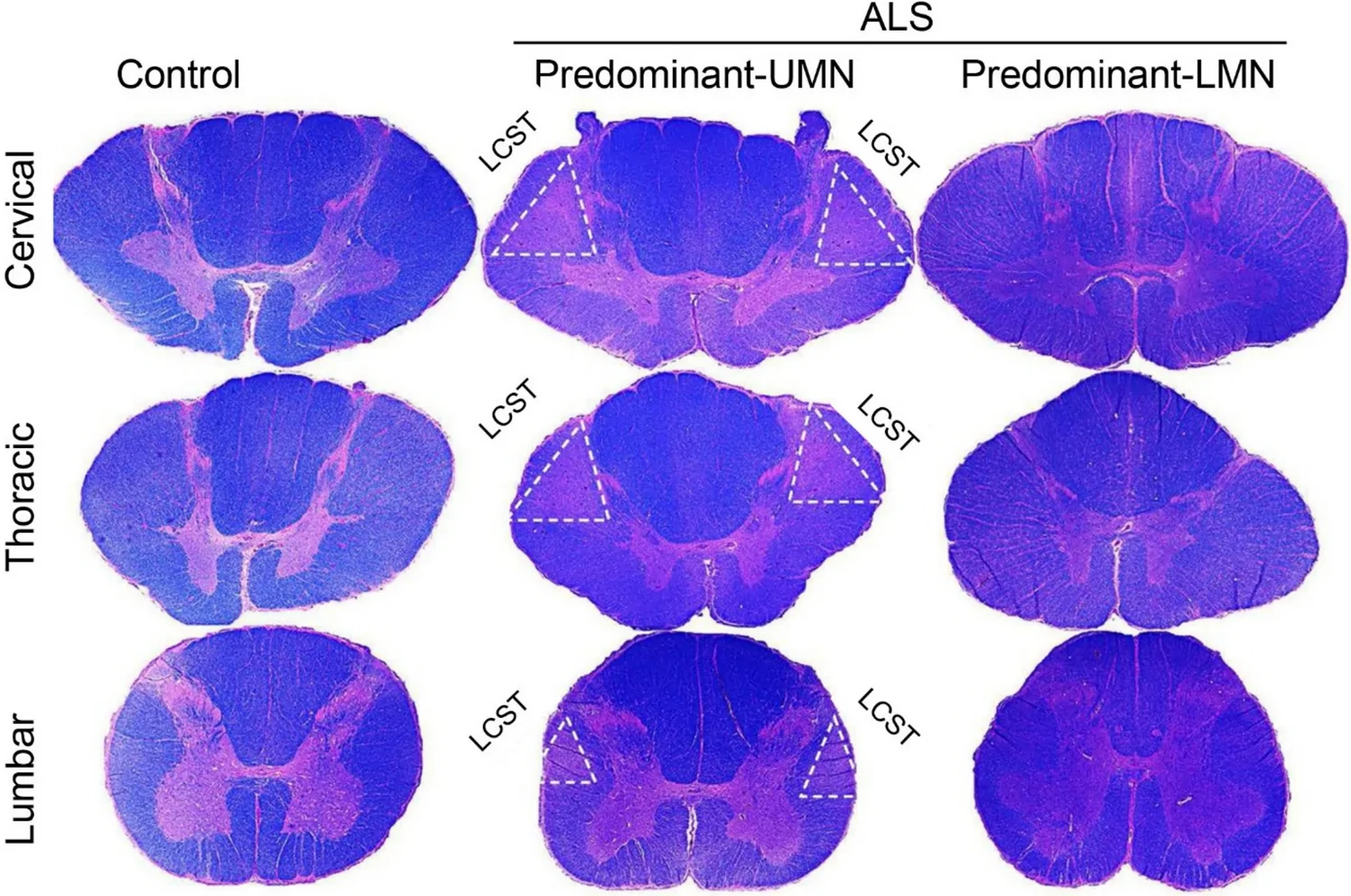
Myelin loss and pathological lesions in the lateral CST at all three levels of the spinal cord (cervical, thoracic, and lumbar) are present only in ALS patients at disease onset with clinically UMN-p symptoms. All spinal cords were analyzed using LFB-PAS tissue staining. The pathological lesion (pink areas) in the lateral CST at all three levels of the spinal cord, composed of myelin loss (fewer blue areas), is present in ALS patients at disease onset with clinically predominant UMN symptoms (6 out of 6 patients). This contrasts with ALS patients at disease onset with clinically LMN-p symptoms (0 out of 6 patients), as well as control patients (0 out of 10 patients) who had no pathological evidence of neurological disease. The images are representative of 6 sALS patients with UMN-p symptoms, 6 sALS patients with LMN-p symptoms, and 10 control patients (Table 1, UIC site). Scale bar = 500 μm. Detailed information is described in the M&M section

We first investigated myelin changes in the CST at three levels (cervical, thoracic, and lumbar) in relation to the clinical characterizations using LFB-PAS tissue staining (Fig. 1). There was distinct loss of myelin in the lateral CST in UMN-p ALS spinal cord compared to LMN-p ALS spinal cords, thus clearly correlating with the clinical phenotyping, and supporting the clinical imputations of UMN degeneration. We found severe myelin loss (stained in pink) in the lateral CST at all three spinal cord levels in all ALS patients with UMN-p symptoms (Fig. 1 and Table 2). In contrast, in ALS patients with LMN-p symptoms, the lateral CST exhibited characteristics similar to other white matter regions at all three spinal cord levels, resembling those observed in control patients (Fig. 1 and Table 2). Notably, when myelin loss and pathological lesions occurred in UMN-p patients, they followed an ‘all or none’ pattern at all three spinal cord levels.

**Table 2:** Incidence of myelin loss in the spinal cord (cervical, thoracic, lumbar)

Autopsy samples	Spinal cord	Descending track (motor axons)		Ascending track (sensory axons)
		Lateral CST	Ventral CST	Dorsal column
Controls (*n* = 10)	Cervical	0/10	0/10	0/10
	Thoracic	0/10	0/10	0/10
	Lumbar	0/10	0/10	0/10
UMN-p ALS (*n* = 6)	Cervical	6/6	6/6	0/6
	Thoracic	6/6	6/6	0/6
	Lumbar	6/6	6/6	0/6
LMN-p ALS (*n* = 6)	Cervical	0/6	0/6	0/6
	Thoracic	0/6	0/6	0/6
	Lumbar	1/6	0/6	0/6

### Axon loss in the lateral CST involves axons of all sizes and is worse distally

**Fig. 2:**
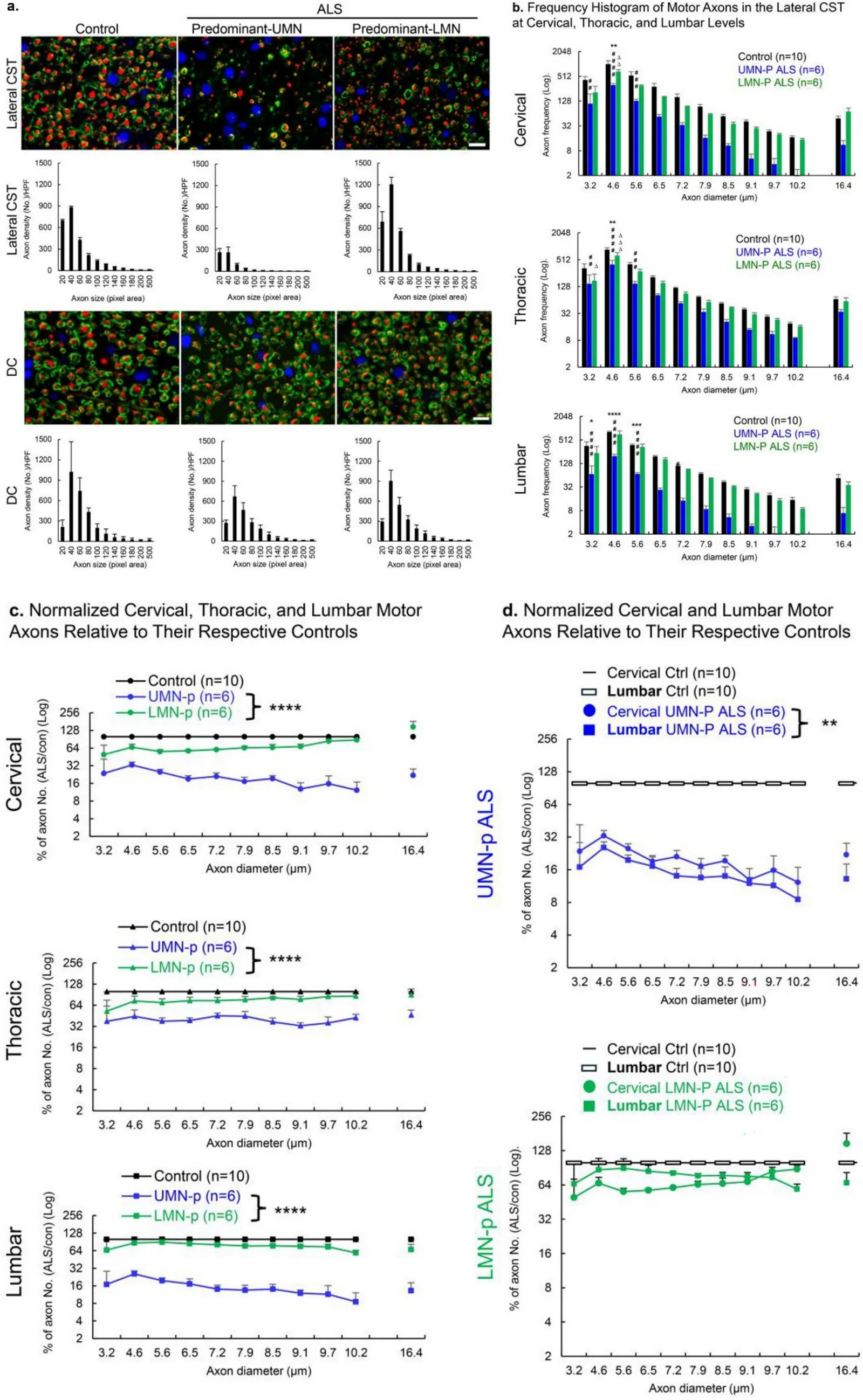
Loss of descending UMN axons in the lateral CST across three spinal cord levels, with more pronounced loss at the lumbar than at the cervical level in UMN-p ALS patients. **a** Representative double immunofluorescence images of the lumbar spinal cord showing the lateral CST and dorsal column in UMN-p ALS, LMN-p ALS, and control cases. Neurofilament (NF; RT97) is shown in red and myelin basic protein (MBP) in green. Axonal density was quantified as the number of axons per high-power field, and histograms illustrate axonal pixel area distribution. Scale bar = 10 µm. **b** Frequency distribution of mean analysis demonstrated a selective reduction of small-diameter axons in UMN-p ALS (*n* = 6) compared with controls (*n* = 10) and LMN-p ALS (*n* = 6) across all spinal cord levels. Statistical analysis was performed using two-way ANOVA (^#^*p* < 0.05, ^##^*p* < 0.01, ^###^*p* < 0.001, ^####^*p* < 0.0001 vs controls; ^∆^*p* < 0.05, ^∆∆^*p* < 0.01, ^∆∆∆^*p* < 0.001 vs controls; *p < 0.05, **p < 0.01, ***p < 0.001, ****p < 0.0001 between ALS groups). **c** Pooled data normalized to control values showed significantly greater axonal loss in UMN-p ALS compared with LMN-p ALS across all axon size categories and spinal cord levels (****p < 0.0001, Student’s *t* test). **d** In UMN-p ALS, axonal loss in the lateral CST was more pronounced in the lumbar than cervical region across all axon size ranges (upper panel), reaching statistical significance (**p < 0.005, Student’s *t* test). No significant regional difference was observed in LMN-p ALS (lower panel). A total of 24 ALS sections (12 per group) and 40 control sections were analyzed per spinal cord level (Table 1). Detailed information is described in the M&M section

It has been previously established that large-diameter axons are selectively lost in the lateral CST, consistent with the selective degeneration of the direct monosynaptic connections between the UMN and LMN [39, 51, 64, 70]. We developed a quantitative method to assess axon size distributions using axon frequency histograms. Initial observations indicated that motor axons in the CST exhibited severe loss across all size ranges in UMN-p ALS, whereas sensory axon size distributions in the DC remained similar among UMN-p ALS, LMN-p ALS, and control individuals at the lumbar spinal cord level (Fig. 2a). We then compared motor axon frequency across three spinal cord levels in all three groups (Fig. 2b). We observed a loss of smaller-diameter UMN axons (3.2–5.6 µm) in the UMN-p ALS group compared with the control and the LMN-p ALS groups across cervical, thoracic, and lumbar levels (Fig. 2b). Although the LMN-p ALS group also showed reductions in smaller-diameter UMN axons relative to controls, this loss was less pronounced than in the UMN-p ALS group (Fig. 2b). To compare the two ALS clinical phenotypes, motor axon counts from each diameter in ALS samples were normalized to their respective controls at each spinal level. The quantification revealed a greater loss of UMN axons across all diameter ranges in UMN-p ALS compared with LMN-p ALS at all three spinal cord levels (cervical, thoracic, and lumbar) (Fig. 2c).

We compared UMN axon loss between the cervical and lumbar spinal cord levels (Fig. 2d). In the UMN-p ALS group, a gradient loss across all UMN axon size ranges was observed in the lumbar relative to the cervical within the lateral CST (Fig. 2d, upper panel), whereas no such gradient was detected in the LMN-p ALS group (Fig. 2d, lower panel). Thus, this suggests that UMN axon loss is worse distally in the axonal projections in UMN-p ALS patients, indicating distal axon loss [15].

### Increased foamy microglia co-localization with myelin and UMN axon loss in the lateral CST of UMN-p ALS patients

**Fig. 3:**
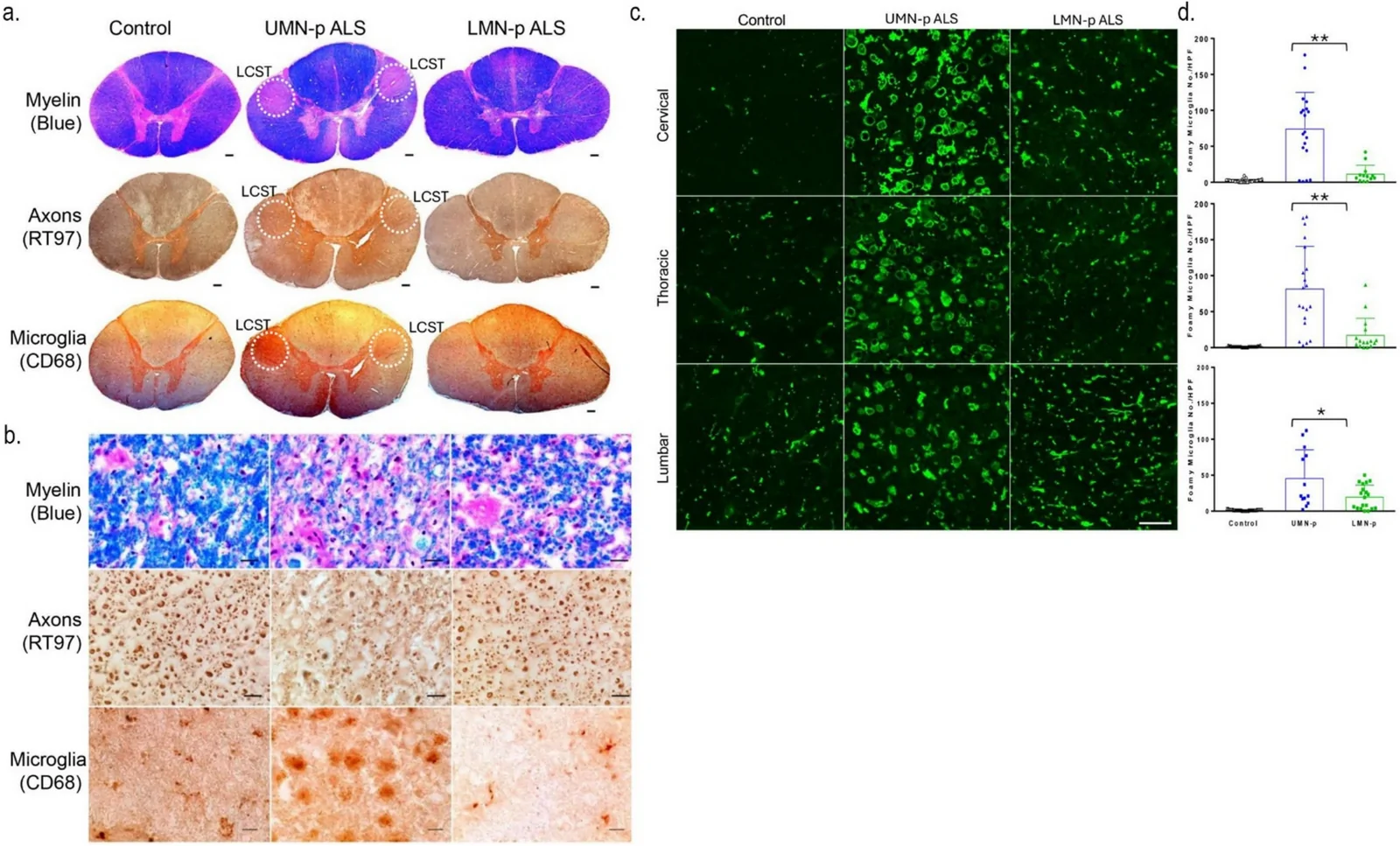
Increased foamy-shaped microglia (innate immune cells) co-localize with loss of myelin and axons in the lateral CST of UMN-p ALS patients. **a** All spinal cords were analyzed for myelin loss, axon loss, and microglial activation using LFB-PAS tissue staining. Myelin loss, axon loss, and microglial activation in the lateral CST were demonstrated by LFB-PAS, and immunohistochemistry using neurofilament (RT-97) and CD68 antibodies (Table 1, UIC site). Scale bar = 500 µm. **b** Myelin loss (pink areas), different sizes of axon loss (RT-97), and activated foamy microglia (CD68 positive cells) shown in the high-powered field of the lateral CST. Scale bar = 20 µm. **c** Increased foamy-shaped microglia in the lateral CST of UMN-p ALS patients. Foamy microglia (Iba1 positive cells) in the lateral CST were quantified by manual counting, as described in the M&M section. **d** The data is presented as the average number of foamy microglia ± SD in the lateral CST from each group of patients. Statistical significance of changes between the groups was assessed using one-way ANOVA. ***p < 0.001

Foamy microglia, characterized by their lipid-laden appearance and lipid droplet accumulation, are a part of loss of axonal integrity and degeneration in the CST [72], contributing to disease progression and motor neuron and axon loss. Given the overall loss of myelin (Fig. 1) and the reduction of all UMN axon sizes in the lateral CST (Fig. 2) of ALS patients with clinical UMN-p signs during disease, we examined whether inflammatory glial activation occurs within these pathological regions. Strong microglia/macrophage signal identified by using CD68- and Iba1- positive staining (Fig. 3a, c) was observed, co-localizing with myelin loss and axon degeneration in the lateral CST of UMN-p ALS patients. High-magnification images of the lateral CST (Fig. 3b) revealed a combination of myelin loss and axonal loss across different sizes, coinciding with foamy microglia occurring exclusively in UMN-p ALS patients. The quantification revealed a significant increase in round, foamy microglia (Iba1+ cells) within the lateral CST at all three spinal cord levels in UMN-p ALS patients compared to LMN-p ALS and control patients (Fig. 3d). Furthermore, no changes were observed in GFAP-positive reactive astrocytes in the lesioned area or DC between the two ALS phenotypes (UMN-p vs. LMN-p) (data not shown). These findings support the well-recognized involvement of foamy microglia in ALS pathogenesis, linking myelin loss to motor axon degeneration and neuroinflammation in the descending UMN axon tract of UMN-p ALS patients, but do not answer whether these changes are primary or reactive.

### Variable loss of motor axon loss in the ventral CST and sensory axons in DC

We also examined the ventral CST, a less well-recognized tract carrying axons from the motor cortex. Although myelin loss was observed (Fig. 1 and Table 2), quantification of axon diameters using the same normalization method yielded variable results that were not consistent with those observed in the CST (data not shown).

**Fig. 4:**
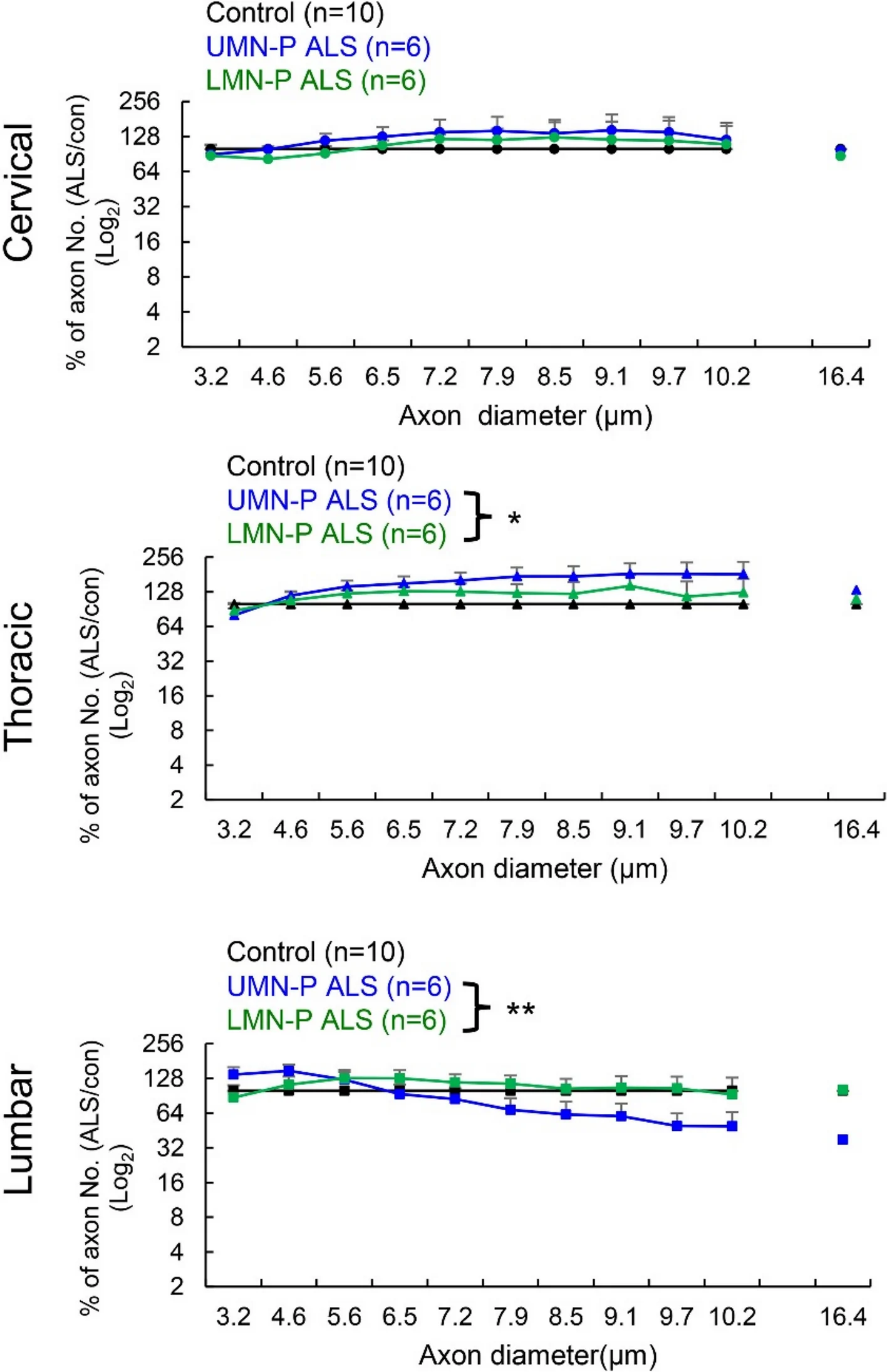
A loss of larger sizes of sensory axons in the ascending tract of the DC was limited to the lumbar spinal cord in ALS patients with UMN-p clinical signs. Mean axon diameter values in the DC for each ALS group at each spinal cord level were normalized to the corresponding control group (*n* = 10; set to 100% at each axon diameter) and compared across axon diameter ranges at cervical, thoracic, and lumbar levels between UMN-p ALS (blue line, *n* = 6) and LMN-p ALS (green line, *n* = 6) subjects (Table 1, UIC site), using the same method described in Fig. 2C. UMN-p ALS patients exhibited significant axon loss at the lumbar spinal cord level compared with LMN-p ALS patients. A total of 18 sections (3 sections per ALS patient group) and 30 sections (10 per control patient) were randomly selected within the DC at each spinal cord level for analysis. Details of the methodology are provided in M&M section. Statistical significance of changes between the groups was assessed using Student’s *t* test. *p < 0.05. **p < 0.005

In contrast, we also examined sensory axons in the DC for comparison with motor axons in the lateral CST. Interestingly, quantification of sensory axon diameters in the DC across the three spinal cord levels revealed a significant loss of ascending axons only in the lumbar region in UMN-p ALS (Fig. 4, bottom panel), while LMN-p ALS samples showed no sensory axon loss at any level. Notably, no significant myelin loss was observed in the DC (Fig. 1 and Table 2), and foamy microglia were absent in the sensory axon tract of the DC in both ALS phenotypes and control patients (Data not shown). This finding implies that sensory axon loss may represent an early stage, as no myelin loss or microglial activation was observed in the DC by tissue staining.

### Axonal TDP-43 was more frequently observed in the CSTs and ventral horn gray matter in sALS cases compared to controls

**Fig. 5:**
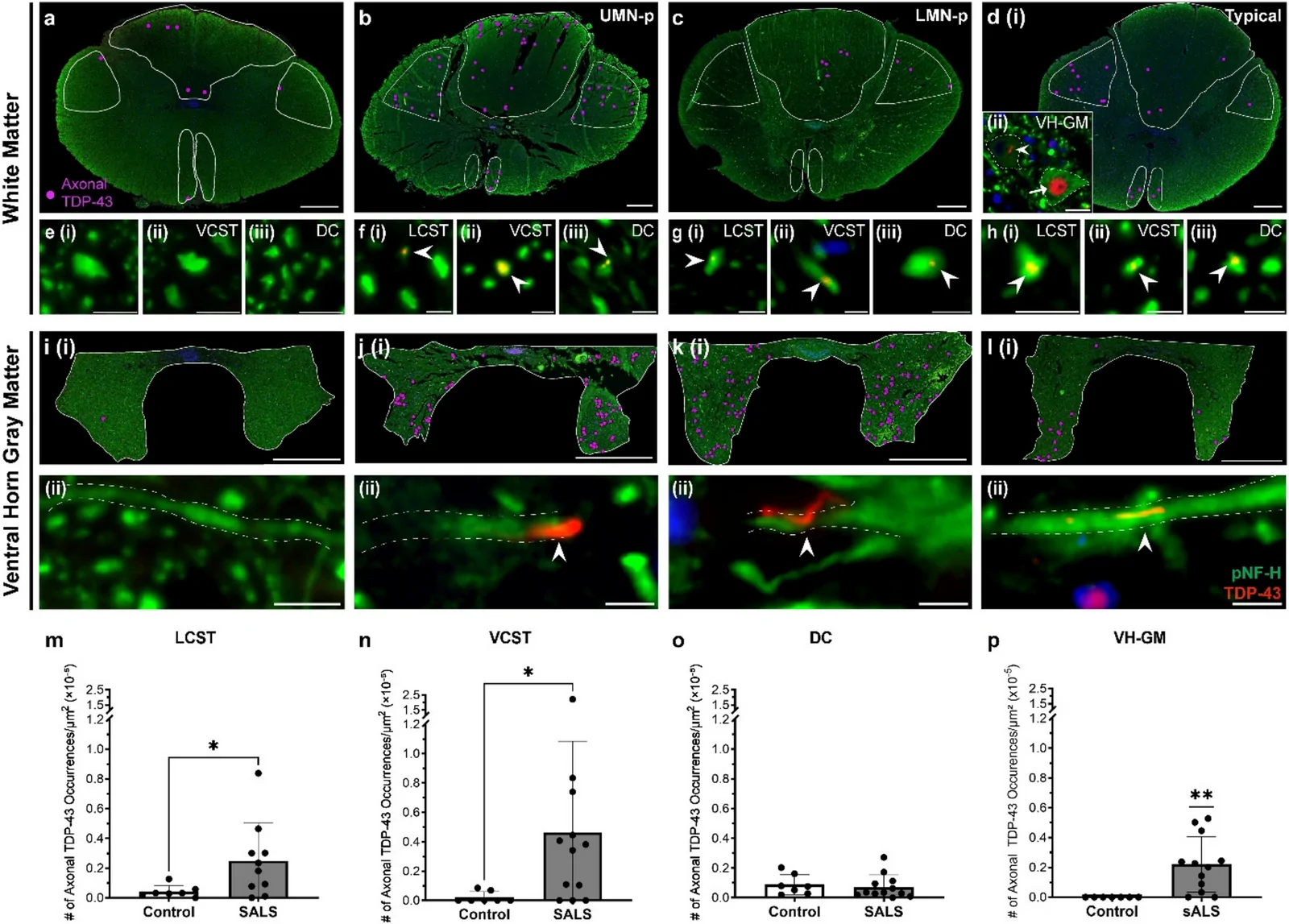
Axonal TDP-43 expression is elevated in the CSTs and ventral horn gray matter in sALS relative to controls. Thoracic spinal cord sections from sALS patients (*n* = 12, Table 1 UCSD site) and controls (*n* = 7, Table 1 UCSD site) were immunostained to detect total TDP-43 (red) and pNF-H (green), a marker of axons. Panels **a–d** Representative images of the axonal TDP-43 distribution pattern in white matter ROIs in control (**a**) and UMN-p (**b**, *n* = 3, Table 1 UCSD site), LMN-p (**c**, *n* = 3, Table 1 UCSD site), and typical (**d**, *n* = 6, Table 1 UCSD site) ALS phenotypes. Each magenta dot is mapped to the location of each axonal TDP-43 occurrence. Scale bar = 1 mm. In Panel **d** (ii), total TDP-43 is shown in the nucleus (arrow) or mislocalized to the cytoplasm (arrowhead) in ventral horn motor neurons, confirming antibody specificity, Scale bar = 20 µm. Panels **e–h**: Representative images of axonal TDP-43 (arrowheads) in the LCST (i), VCST (ii) and DC (iii) for control (**e**) and each ALS phenotype: UMN-p (**f**), LMN-p (**g**), and typical (**h**). Scale bar = 5 µm. Panels **i–l** (i): Representative images of the axonal TDP-43 distribution pattern in the VH-GM in control (**i**) and UMN-p (**j**), LMN-p (**k**), and typical (**l**) ALS phenotypes. Each magenta dot is mapped to the location of each axonal TDP-43 occurrence. Scale bar = 1 mm. Panels **i–l** (ii): Representative images of fibril-type axonal TDP-43 (arrowheads) in the VH-GM for control (**i**) and each ALS phenotype: UMN-p (**j**), LMN-p (**k**), and typical (**l**). Axons are outlined by dotted lines. Scale bar = 5 μm. Panels **m–p** Quantification of axonal TDP-43 frequency per 10^−5^ × μm^2^ in the LCST (**m**), VCST (**n**), DC (**o**) and VH-GM (**p**). Axonal TDP-43 frequency is notably increased in the lateral (**m**, *p* = 0.04) and ventral (**n**, *p* = 0.01) CSTs and VH-GM (**p**, *p* = 0.001), but not in the DC (**o**, *p* = 0.54) in sALS cases compared to controls

Mislocalization from the nucleus, cytoplasmic aggregation and phosphorylation of TDP-43 is the hallmark of ALS neuropathology in the cell bodies. Aggregation of phosphorylated TDP-43 has been identified in axons in LMNs [3, 10, 11, 43, 49, 57]. Phosphorylated TDP-43 is rarely observed in the lateral CST consistent across three independent laboratories (Ravits, Loeb and Stokin, data not shown) and it does not appear to have been previously reported [11]. In order to study this further, we measured TDP-43 in the CSTs and the ventral horn in thoracic spinal cords from three phenotypes of sALS patients—UMN-p, LMN-p and typical ALS and in controls (Table 1). While we did not observe abnormal TDP-43 aggregates, when we measured total TDP-43 in axons by colocalizing with pNF-H in this way, it was quantitatively increased in the lateral CST (Fig. 5m, *p* = 0.04), as well as in the ventral CST (Fig. 5n, *p* = 0.01) in sALS spinal cords. No significant differences in total TDP-43 levels were observed in sensory axons of the DC between sALS (Fig. 5o(iii), *p* = 0.54) and control spinal cords (Fig. 5p). Total TDP-43 (red) was also detected in LMN nuclei, with and without cytoplasmic mislocalization (Fig. 5d(ii)), which confirmed the sensitivity of the antibody as previously reported [1]. Notably, axonal TDP-43 fibrils were observed in the ventral horn gray matter in sALS spinal cords (Fig. 5j–l), colabeled with axons (pNF-H, green), and were absent in controls (Fig. 5p, *p* = 0.001), consistent with other reports in LMNs [3, 10, 11, 43, 49, 57]. When comparing regional TDP-43 differences between the ALS subtypes, only the typical sALS group shows significantly more inclusions in gray matter compared to controls. (Kruskal–Wallis, *p* = 0.02, data not shown) Thus, TDP-43 is measurably increased in UMN axons in the descending CSTs but may be different in LMN axons in the peripheral nervous system and ventral horn gray matter, suggesting molecular mechanisms of TDP-43 may be different in UMNs compared to LMNs.

## Discussion

### “Lateral sclerosis”: myelination, axonal degeneration and neuroinflammation in the lateral CST

By focusing on the extremes of clinical phenotypes, we were able to correlate clinical signs of UMN degeneration with the neuropathological changes in the lateral CST, the axon tracts of the UMN, something difficult to do by counting neurons in the motor cortex or quantifying TDP-43 neuropathology. The large-diameter axons originate from Betz cells in the primary motor cortex [50], and form the longest axons in the CST [50], and are known to be preferentially involved in ALS [39, 51, 64, 70]. Our findings extend these observations and find that involvement progresses to smaller axons as well. This supports the complexity of the UMN motor tracts [23, 45, 56] and significant loss of M1 fibers in the ALS brain, as reported only in the NODDI imaging study [26, 35]. The findings also indicate extensive myelin loss and inflammation (Fig. 1).

We speculate that the spatial differences between axon degeneration and demyelination/microgliosis may reflect distinct pathological processes during disease progression. Early UMN axon degeneration likely triggers microglial activation, promoting the clearance of degenerating axons and myelin debris through phagocytosis and axonal pruning. As degeneration progresses, the continued accumulation of myelin debris and ongoing axonal loss may overwhelm the phagocytic capacity of microglia, leading to the formation of lipid-laden, foamy microglia. Consequently, although axon degeneration exhibits a rostro-caudal gradient, demyelination and microglial activation may become more uniformly distributed throughout the CST as the disease reaches its end-stage. This interpretation is consistent with the notion that microgliosis and myelin loss represent downstream responses to chronic axonal degeneration rather than simply mirroring the regional severity of axon loss.

It is certainly possible that axons undergoing distal degeneration have already experienced proximal myelin loss. However, the current study was limited to assessing myelin integrity using immunofluorescence and immunohistochemistry, which do not provide sufficient resolution to determine whether proximal demyelination precedes, accompanies, or follows distal axonal degeneration. Future studies employing higher-resolution techniques, such as electron microscopy, will be necessary to define the temporal and spatial relationship between axonal degeneration and myelin loss along the corticospinal tract. Such studies may also help clarify whether proximal myelin abnormalities represent an early pathological event or develop secondary to ongoing axonal degeneration.

### Lateral sclerosis is a distal axonopathy

In our study, we found evidence indicating that degeneration of UMNs is a distal axonopathy; that is, UMNs are “dying back”, a finding previously discussed by others in sporadic ALS [24] and in the context of mutant SOD1-associated ALS neuropathology and experimental mouse models [21, 26] (Table 3). In a neuropathological study of sporadic ALS, C. Davison [24] observed that lateral sclerosis is characterized by degeneration of UMN fibers, most pronounced in the distal segments of the corticospinal tract, a finding also recently re-introduced [21]. In the neuropathological study of mutant SOD-associated ALS, degeneration was observed in the corticospinal tracts but not the cortical neurons, an especially interesting finding in light of the belief that SOD1 mutations in ALS affect LMNs more than UMNs clinically [37]. In the study of the experimental mouse model of mutant SOD1-associated ALS, dying back was established in LMNs, studying the neuromuscular junction, ventral roots, and anterior horn of the spinal cord [29]. This study included one human autopsy in which the LMN was studied by way of the spinal anterior horn and the neuromuscular junction, but the axons of the UMN were not discussed.

**Table 3:** Clarification of ‘dying-back’ and ‘dying-forward’ as applied to ALS

	Definition	UMN	LMN
Dying back	“Dying back” is a term traditionally used to convey a pattern of axon loss that is length-dependent, presumably from the inability of the cell body to maintain the full length of its axon	UMN axons descend in the cortico-spinal tracts, and length-dependent axon loss (“dying back”) manifests as worse caudally than rostrally, causing clinical deficits to be greater in the legs than in the arms An example is hereditary spastic paraparesis	LMN axons travel in roots and peripheral nerves, and length-dependent axon loss (“dying back”) manifests as retraction from the NMJ and denervation of muscle that is worse in distal muscles than proximal ones Examples are peripheral polyneuropathies, which have a “stocking-glove” pattern This contrasts with *segmental* patterns, which are based on spinal neuron and root anatomy, such as in spinal muscular atrophy or Guillain-Barré syndrome, or diabetic poly*radiculo*pathies
Dying forward	“Dying forward” is a phrase coined specifically for ALS mechanisms, speculating that the motor cortex and UMN have primacy in driving the degeneration of LMNs by an antegrade transneuronal process The term was deliberately chosen to contrast with the well-recognized “dying back” term (above), unfortunately leading to “dying back” sometimes being misconstrued as a retrograde transneuronal mechanism of motor neuron degeneration	The dying forward hypothesis poses that the failure in ALS is in the cortex, which is driving LMN degeneration in the brainstem and spinal cord; in effect, UMNs are killing LMNs The explanations for the heterogeneity of ALS phenotypes, especially the variation of somatic sites at onset or variable involvement of UMN and LMN, by a “dying forward” mechanism, are not stated	In the dying forward hypothesis, LMNs are victims of UMNs; LMNs degenerate secondary to or as a consequence of UMN factors
Prion-like propagation	“Prion-like propagation” is a term used in neurodegenerative disease biology, including ALS, to speculate that toxicity is linked to factors such as protein folding and templating spreading in neuronal networks, which in ALS is the motor system, both UMNs and LMNs When applied to ALS mechanisms, one important distinction with the term “dying forward’ is that it is agnostic about primacy of UMNs or LMNs	Toxic factor (eg, TDP-43 misfolding) is a stochastic molecular event that is randomly located anatomically, variably distributed between UMNs and LMNs, and spread by way of cell-to-cell propagation (contiguously or trans-axonal or in CSF) Once triggered, UMN and LMN degeneration propagate independently anatomically	Heterogeneity of clinical phenotype patterns can be explained based on stochastic anatomic and molecular features that are propagating The observation that the initial UMN and LMN clinical deficits appear in the same somatic region suggests that the trigger is within the LMN-UMN network innervating that somatic locus but they propagate independently

The concept of a “dying back” axonopathy emerged from classic neuropathologic studies of nerve diseases, in which degeneration was observed to begin in the distal portions of long axons and progress proximally toward the cell body [16, 65]. The term “central-peripheral distal axonopathy” was used to describe a unified pattern of length-dependent degeneration affecting both peripheral and central projections [66]. Subsequent mechanistic work linked these observations to impairments in axonal transport and cytoskeletal integrity, providing a biological basis for the vulnerability of distal axons [33]. Collectively, these studies established distal axonopathy as a fundamental and reproducible pattern of neurodegeneration and remain a cornerstone for understanding length-dependent vulnerability in the nervous system.

The classical examples of length-dependent dying back are peripheral polyneuropathies, which typically have a stocking-glove pattern of deficits. When peripheral neuropathies are not length-dependent, the term neuronopathy is often invoked to distinguish this from axonopathy, and the pattern is often referred to as segmental or radicular. Examples of such segmental or radicular patterns in the motor system are spinal muscular atrophy (which is a disease of the spinal motor neurons), Guillain–Barré syndrome (which is a disease causing demyelination especially involving motor nerve roots), and a rare complication of diabetes called diabetic polyradiculopathy (which is distinct from the common diabetic polyneuropathies, which are indeed dependent on length).

This concept of dying back or distal axonopathy is complex and difficult to characterize in ALS because the motor system has two tiers. (Table 3). The UMNs travel in the corticospinal tracts and synapse on lower motor segments. The length-dependent clinical pattern produced by their degeneration affects the legs more than the arms. By contrast, the LMNs travel through the motor roots and peripheral nerves and synapse on muscle fibers. The length-dependent clinical pattern produced by their degeneration affects distal muscles more than proximal ones within the same limb (both arms and legs). Since ALS begins focally in any somatic region, and variably involves UMN and LMN degeneration, clinical patterns of length-dependent axonopathy are easily masked from detection. Further, length-dependent abnormalities are difficult to detect when considering whether the axons are part of a monsynaptic or polysynaptic pathway. The fact that the largest diameter axons in the corticospinal tract display the most involvement in previous studies [51, 64, 70] is consistent with a dependence on length—the largest diameter axons are monosynaptic, and since monosynaptic, also longest. The length of axons in polysynaptic pathways, by contrast, is shorter, but their distal ends are essentially unknown, depending on the number of neurons that comprise the pathway. Our quantitative neuropathological study, which examines the extremes of clinical phenotypes, has unmasked dependence on length and shown involvement of polysynaptic as well as monosynaptic components of the UMN tier.

Dying back, distal axonopathy, or length-dependent axonal pathology, are historically descriptive terms for distal-to-proximal axonal degeneration and are noncontroversial. It seems likely that they are caused by a proximal failure in the neuron, likely in the cell body, and that distal axons are affected secondarily. In favor of this is the recently emerging evidence of TDP-43 loss-of-function in STMN2 [42, 48], UNC13A [13, 47], and many other transcripts [75]. When TDP-43 mislocalizes from the nucleus, genes that are modulated by it may be reduced by way of polyadenylation or nonsense-mediated decay. STMN2 plays an important role in axon maintenance and regeneration, and UNC13A plays an important role in neuromuscular junction function. Thus, loss of TDP-43 function in the nucleus leads to failure distally. This has been mainly studied in LMNs, and the role of TDP-43 loss of function in UMNs remains a relative blind spot and easily overlooked, for example, in iPSC models of motor neurons and omics studies of human brain tissue. TDP-43 may function differently in UMNs than in LMNs.

The term “dying forward” in the ALS literature has parlayed on the terms of dying back and in so doing, can be easily confused. The main source of confusion is mixing patterns (distal dying back) with mechanisms (dying forward, which is speculative). The first use of the term “dying forward” in ALS was in 2001 [27] in one of the many iterations of a hypothesis that first appeared in 1992, positing the motor cortex as the driver of ALS [26]. According to this hypothesis, ALS is driven by transneuronal antegrade transmission of a toxic factor from the motor cortex. The term was chosen to contrast with dying-back, a pattern well known to neurologists. The dying forward hypothesis has more recently been referred to as “corticofugal axonal spread” [9]. This hypothesis has served as a basis for many observational papers related to neuroexcitatory toxicity and neurophysiological studies involving magnetic stimulation of the cortex, but only rarely supported experimentally [71]. The hypothesis has been criticized for many reasons [38, 40, 52, 68], recently, for its inability to explain the heterogeneity of motor phenotypes [55]. While dying forward is hypothesizing a mechanism, dying back is not—that is, it does not mean that the axons are killing the neurons (sick neurons are going to have sick axons, and this is going to be worse distally) or that LMNs are killing UMNs. That one motor tier (eg UMN) is more important in pathogenesis than the other (eg, LMN) or vice versa is specultative [4, 25, 27].

Neuroinflammation contributes to motor neuron degeneration and disease progression in ALS, making it a potential target for therapeutic interventions [12, 34, 46, 74]. We [62] and others [12, 36] previously reported that microglial pathology, as indicated by CD68 and Iba1 expression, is significantly more extensive in the CST of ALS cases. The extent of microglial activation and myelin loss in the CST correlates with disease progression and the severity of UMN deficits [12, 36, 62]. Here, we present findings demonstrating a combined loss of myelin (Fig. 1) and axons of all sizes in the lateral CST (Fig. 2), which coexists with a unique subtype of activated inflammatory microglia/macrophages (Fig. 3). While foamy phagocytes have been primarily studied in multiple sclerosis [32, 72], our data suggest that they also play a significant role in ALS pathology. These foamy microglia characterized and subtyped by immunohistochemical methods, may contribute to disease progression through their phagocytic activity and inflammatory properties, or they may be secondary effects clearing the deteriorating axons.

We propose that homeostatic CNS microglia transform into inflammatory foamy microglia in response to axonal loss and myelin loss in the lateral CST at early disease stages in UMN-p ALS patients. Additionally, foamy macrophages were frequently observed in areas of motor neuron loss and gliosis within the anterior horn of the spinal cord, suggesting that peripheral innate immune cells may also interact with CST pathology [20]. The extremely tight spatial correlation between foamy microglia and regions of the CST with the most severe myelin and axonal loss could be compatible with these cells representing either an upstream pathogenic driver or a secondary phagocytic and remodeling response to preexisting axonal degeneration. Understanding these neuroinflammatory interactions could provide valuable insights into developing targeted therapies and biomarkers, paving the way for more precise ALS treatments.

On the other hand, clinically, some ALS patients with UMN-p signs may exhibit slower disease progression and longer survival; however, in this series, they showed the most dramatic CST myelin and axonal loss, accompanied by prominent microglial accumulation. Possible explanations include a prolonged preclinical period characterized by slow but relentless CST degeneration, and the capacity of the CST to accumulate extensive structural damage without a proportionate impact on short-term survival when LMNs are relatively spared. Our clinical–neuropathological findings in the two specific clinical phenotypes suggest that axons of all calibers support distal vulnerability in lateral sclerosis in UMN-p ALS samples; however, given the small sample sizes in each phenotype group, the current data could not distinguish between a primary “dying-back” process and advanced tract involvement in end-stage disease.

Furthermore, at disease onset, most patients exhibit left-to-right asymmetry in their clinical symptoms. However, as disease progresses most patients become diffuse and symmetrical and, in the end-stage autopsy samples, we did not observe a clear or consistent left-to-right asymmetry in CST pathology, nor did we find a consistent correlation between pathological asymmetry and the initial clinical presentation. At the end stage of the autopsy samples (after individual diagnosed ALS disease onset 0.9–12 years, Table 1), we did not observe a clear or consistent relationship between the site of clinical symptom onset and the extent of neurodegeneration, demyelination, or microgliosis in either ALS subtype. In the end-stage autopsy samples, we found that patients with bulbar-onset ALS exhibited CST axon loss in the lumbar spinal cord and the cervical spinal cord. This observation suggests that degeneration of UMN axons projecting to the lumbar spinal cord progresses independently of the initial site of somatic involvement at disease onset. There are two independent axes of UMN degeneration: one is the site of onset and the other is the degree of UMN degeneration with respect to the degree of LMN degeneration.

In addition, the classical mixed-presentation ALS cases (with both UMN and LMN clinical involvement) were not included in the present study because they have recently been reported in our publication in Scientific Reports [21]. In that study, end-stage pathology of the mixed-presentation ALS cases showed a pattern of CST UMN axon loss that was similar to that observed in the UMN-p group in the current study. This finding is consistent with the presence of significant UMN clinical involvement in mixed-presentation cases.

### Observations in the ventral CST and DC

Our studies shed light on two other spinal tracts, the ventral CST and the DC. The ventral CST, which contains uncrossed fibers and only comprises approximately ten to twenty-five percent of corticospinal fibers [61, 67]. These anatomical variations may explain the inconsistency with which myelin and axon loss were observed in the ventral CST in the lumbar spinal cord of UMN-p ALS spinal cord (Fig. 1 and Table 2). Due to variability and inconsistency between the two normalization methods—originally optimized for the lateral CST—we were unable to draw definitive conclusions regarding axon changes in the ventral CST although there is trend that axon loss in the ventral CST fully contributes to ALS pathology [6, 14, 37, 51, 53, 73].

Interestingly, we found that sensory axon loss in DC predominantly affects lumbar regions (Fig. 4). To the best of our knowledge, this is the first report on sensory axon loss in the lumbar spinal cord using systematic quantification methods, comparing three spinal cord levels in UMN-p ALS to LMN-p ALS relative to controls. Other studies have reported sensory axon loss in the DC of the lumbar spinal cord as part of the broader multisystem involvement in ALS, supporting the inclusion of sensory neuropathy among the non-motor manifestations of the disease [8, 41, 58]. While sensory involvement is not a clinical feature of the disease, occasional sensory symptoms are observed. It is possible that the mechanisms of sensory axon degeneration in the DC (Fig. 4) are different from motor axon degeneration in the lateral CST (Fig. 2), as no myelin loss (Fig. 1 and Table 2) and no foamy microglia (Fig. 3) were observed in the DC. Further understanding of the multisystem nature of ALS is an important future area to be studied.

### Increased frequency of axonal TDP-43 instances in the CSTs and the gray matter of sALS spinal cords

Axonal TDP-43 has been observed in the human ALS motor cortex and medulla [11], ALS patient-derived induced pluripotent stem-cell (iPSC) MNs [3], human and mouse ALS peripheral nerves [57], and ALS transgenic mouse models [3]. To our knowledge, this is the first reporting of axonal TDP-43 pathology in both the CST and gray matter of the spinal cord in sALS (Fig. 5). Axonal TDP-43 pathology along the CST has been proposed to contribute to ALS pathogenesis by mediating trans-axonal spread of disease between the motor cortex and spinal motor neurons; however, in patients this has largely been inferred rather than directly demonstrated. Our findings demonstrate increased total TDP-43 signal within CST axons and LMN axonal regions, rather than classic phosphorylated inclusions; the functional significance of this subtle increase remains speculative. These observations may be interpreted as evidence of compartment-specific TDP-43 dysregulation that differs between axons and cell bodies of UMNs and LMNs, and as a stimulus for future mechanistic studies.

To our knowledge, axonal phospho-TDP-43 pathology within the human lateral CST has not been previously reported. Most previous studies have focused on phospho-TDP-43 aggregates in neuronal cell bodies and glial cells rather than within axons. Based on our analyses, phospho-TDP-43 immunoreactivity was only rarely observed [OR NOT OBSERVED AT ALL] in the lateral CST by either immunohistochemistry or immunofluorescence. This finding was independently confirmed in three separate laboratories, but the staining was too infrequent and inconsistent to permit meaningful quantitative analysis; therefore, these data were not included in the manuscript. Although Western blotting could, in principle, provide quantitative information, and we have been working on this unsuccessfully thus far—the approach is technically challenging for the lateral CST because it requires precise microdissection of this relatively small white matter tract from individual postmortem spinal cord specimens, limited amount of tissue, and purification of protein from soluble and insoluble fractions. We are continuing to work on this.

## Conclusions

Our findings demonstrate that "lateral sclerosis"—a key feature of ALS—reflects multiple pathological changes: UMN axonal degeneration, myelin loss, inflammatory foamy microglia and length-dependence (‘dying back’). The presence of total TDP-43 was observed in UMN axons in both UMN-p and LMN-p ALS phenotypes. This co-occurrence suggests that neuro-glial interactions within the lateral CST underlie disease progression and the importance of studying UMN from LMN axon biology in ALS pathogenesis [28].

## Abbreviations

ALS: Amyotrophic lateral sclerosis
UMN: Upper motor neuron
LMN: Lower motor neurons
UMN-p: Predominantly upper motor neuron phenotype
LMN-p: Predominantly lower motor neurons phenotype
CST: Corticospinal tracts
DC: Dorsal column
TDP-43: TAR DNA-binding protein 43
Dying back: Distal axonopathy
